# Structural, magnetic, and gigahertz-range electromagnetic wave absorption properties of bulk Ni–Zn ferrite

**DOI:** 10.1038/s41598-021-88930-0

**Published:** 2021-05-04

**Authors:** M. Derakhshani, E. Taheri-Nassaj, M. Jazirehpour, S. M. Masoudpanah

**Affiliations:** 1 Department of Materials Engineering, Tarbiat Modares University, 14115-143, Tehran, Iran; 2 Department of Electroceramics and Electrical Engineering, Malek Ashtar University of Technology, Tehran, Iran; 3 School of Metallurgy and Materials Engineering, Iran University of Science and Technology (IUST), Narmak, Tehran, Iran

**Keywords:** Engineering, Materials science

## Abstract

Nickel–zinc ferrite (Ni_0.5_Zn_0.5_Fe_2_O_4_) powders were prepared by the conventional solid-state route and sintered at 1100 and 1300 °C for utilization as a tile electromagnetic wave absorber. Structural, magnetic, and microwave absorption properties were investigated by characterization techniques of X-ray diffraction, thermogravimetric analysis, Raman spectroscopy, electron microscopy, vibrating sample magnetometry, and vector network analyzer. The samples sintered at 1300 °C showed high magnetic saturation of 87 emu/g and low coercivity of 4 Oe. Electromagnetic investigations exhibit high reflection losses up to − 48.1 dB at certain high and low gigahertz frequencies, as clearly depicted in the 3D contour plot. The optimized condition between reflection loss, thickness, and bandwidth revealed a reflection loss of about − 36.1 dB at the matching thickness of 3.7 mm for the X-band. Furthermore, the effective working bandwidth at − 10 dB was up to ~ 7.1 GHz for the minimum thickness of 4.3 mm, which thoroughly covered the C-band. The microwave absorption performance of the well-sintered Ni–Zn ferrite was attributed to the incorporation of dielectric and magnetic loss mechanisms in which the magnetic part prevails.

## Introduction

Wireless communication transmitting information from one point to another, without using any connection like wires, cables, or any physical medium, is widely used in mobile phones, GPS receivers, remote controls, and Wi-Fi, owing to its inexpensive, mobility, easiness, and reliability^1,2^. However, there are few drawbacks such as interference, security, and probable health issues for wireless communications^3–5^. The interference of electromagnetic (EM) waves from various devices can be led to high amounts of noise in connections by undesired constructive/destructive ways^6–9^. Among various methods, the shielding of electronic devices by microwave absorbers is an attractive and practical method to be safe against electromagnetic interferences and brings good electromagnetic compatibility (EMC) for the operating devices^10,11^. An ideal absorber material for electromagnetic interference (EMI) shielding should have high dielectric and magnetic losses, chemical stability, low-cost, and lightweight^12,13^.

Dielectric^14^, magnetic^15^, and carbon-based^16,17^ materials could be utilized as EM wave absorbers in order to attenuate the distinct frequency range and reducing the interference caused by airborne connections^18^. In addition to the attenuation ability, which is related to dielectric and magnetic losses, another challenging issue is that an absorber material should fulfill the impedance matching condition $$\left( {z = \sqrt {{\raise0.7ex\hbox{$\mu $} \!\mathord{\left/ {\vphantom {\mu \varepsilon }}\right.\kern-\nulldelimiterspace} \!\lower0.7ex\hbox{$\varepsilon $}}} \sim1} \right)$$ to receive incident EM waves with the lowest reflectivity. In most cases, absorber materials possess low permeability and high permittivity, while magnetic spinel ferrites have the competency of good impedance matching according to their higher permeability over other absorbers. Spinel ferrites (MFe_2_O_4_, M = Co, Ni, Mn, Zn, Fe, Cu) as soft magnetic materials can be used for diverse applications such as home and industrial electronic devices^19,20^, biomedicals^21–23^, catalysts^24,25^, and further in EMI shielding as absorber materials because of their relatively high dielectric/magnetic loss, excellent chemical stability, and low costs^26,27^. Nickel–Zinc ferrite shows better EM wave absorption performances compared to other spinel ferrites due to higher resistivity, high saturation magnetization, and high permeability^28–30^. The spinel Ni–Zn ferrite has a cubic crystal structure with the space group of Fd3m in which the cations are distributed between tetrahedral (A) and octahedral [B] sites^12^. The occupation of (A) sites and [B] sites strongly depends on the ionic radius, crystal field, electron configuration, and ionic polarization of cations^31,32^. Accordingly, the Zn^2+^ cations are usually preferred to occupy (A) sites because of crystal energy stabilization, while the octahedral [B] sites are appropriate for Ni^2+^ cations^33,34^. The Fe^3+^ cations can distribute between (A) sites and [B] sites due to the low crystal field energy stabilization^35^. The higher saturation magnetization (Ms) of Ni_0.5_Zn_0.5_Fe_2_O_4_ ferrites than that of NiFe_2_O_4_ ferrite is due to the higher difference of magnetic moments between (A) sites and [B] sites, which is induced by Zn^2+^ doping^36,37^.

Up now, alongside the reports on the electromagnetic properties of Ni–Zn ferrite in the low frequency ranges^38–40^, Huang et al.^35^ studied the magnetic and microwave absorption properties of paraffin/Ni–Zn nanofiber and expressed a magnetic saturation of about 77 emu/g and a reflection loss (RL) of − 14.1 dB at 10.9 GHz. Aggarwal and Narang^41^ investigated the 8.2–12.4 GHz range electromagnetic properties of the nickel–zinc ferrite prepared by the sol–gel route and reached to minimum reflection loss of − 17.5 dB with the effective absorption band length of 3.1 GHz. Mustaffa et al.^42^ scrutinized the microwave absorption properties of MWCNT/Ni–Zn composite and gained an RL = − 19.3 dB with the bandwidth of 1.24 GHz at X band frequency.

In microwave absorber application, spinel ferrite powders were usually prepared by a solid-state^43,44^ or a wet chemical route^45,46^ and mixed with different amounts of a suitable non-conductive or conductive polymer as the dispersant medium, and the composite mixture can be applied as coating^47,48^. The polymer/ferrite composites are disposed to destruct in harsh environments and do not have good durability as a permanent coating^49^. Moreover, it was mostly reported that the EM wave absorption performance might improve by maximizing the Ni–Zn ferrite content^50,51^, but the limitations of adding high amounts of ferrite powders besides the lack of integrity of the polymer-based coatings^49^, lead our team to investigate the microwave absorption properties of sintered Ni–Zn ferrite in a wide 1–18 GHz frequency range for utilization as tile absorber. Also, Naidu^52^ showed that bulk Ni–Mg ferrite has better microwave absorption properties than that of Ni–Mg nanoferrites. In the present work, bulk Ni_0.5_Zn_0.5_Fe_2_O_4_ samples were fabricated by the conventional ceramic method, and the structural, microstructural, magnetic, and microwave absorption properties were studied as a function of sintering temperature.

## Experimental procedure

**Figure 1:**
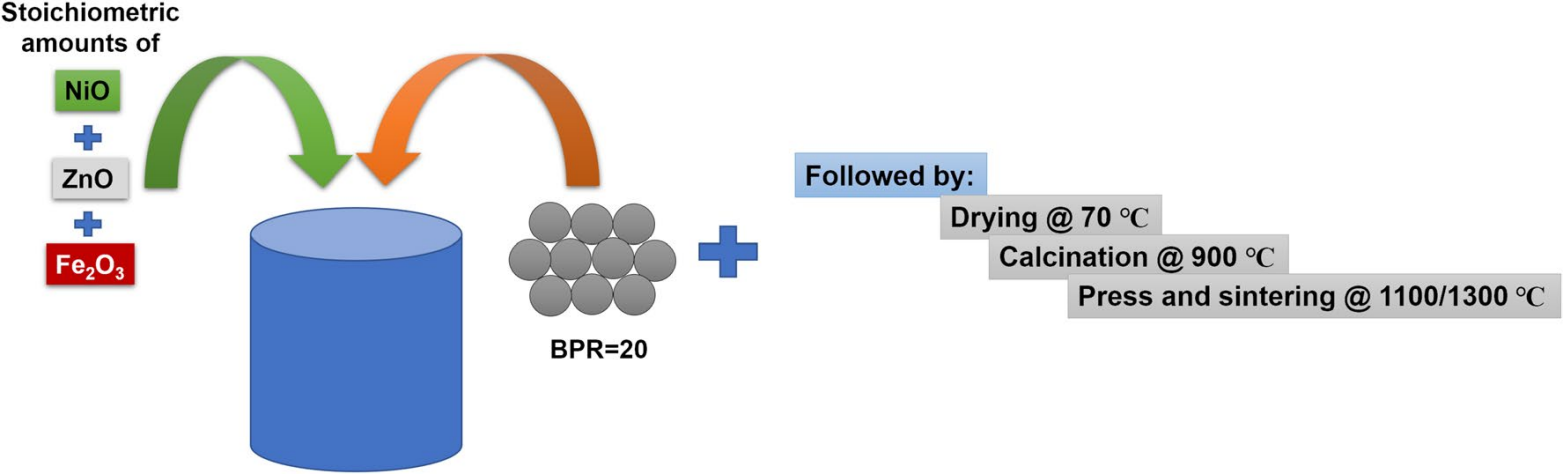
Schematic of the samples synthesis route.

The Ni–Zn ferrite powders were synthesized by the conventional solid-state method. Analytical grade of Fe_2_O_3_, ZnO, and NiO powders in stoichiometric amounts (1:0.5:0.5) was mixed by using a planetary ball mill (Sanat Ceram, Iran) in a wet ethanol medium. The milling rotational speed was set at 200 rpm for 7 h with the ball to powder ratio (BPR) of 20. The obtained mixture was dried in the oven at 70 °C and then calcined at 900 °C in the air atmosphere. The calcined powders were ground again to break the large agglomerates. The as-calcined NiZn ferrite powders were uniaxially pressed into both disk-shaped (d = 10 mm) and toroidal ones (d_in_ = 3 mm and d_out_ = 7 mm) under the pressure of 250 MPa. The compacted bodies were sintered at 1100 and 1300 °C for 2 h in the air atmosphere. The schematic of the synthesis procedure is shown in Fig. 1.

Thermogravimetric analysis was operated with a simultaneous thermal analyzer (BAHR 504) from room temperature up to 1500 °C with the same condition used for calcination and sintering to show the thermal stability of the samples. The structural properties were characterized using an Ultima IV X-ray diffractometer (Rigaku, Japan) with Cu Kα (λ = 1.5406 A) radiation. Crystal structure analysis of the XRD patterns was done using Highscore plus software, and the lattice parameter and cation distribution of the samples were calculated by the Rietveld refinement method. The nickel–zinc ferrite belongs to the spinel Fd3m space group; therefore, oxygen positions (x = y = z) were taken as free parameters, while other atomic fractional positions were held as fixed. Moreover, lattice constants, isothermal parameters, occupancies, scale factors, and shape parameters were also taken as free parameters. Refinement steps were continued until the goodness of fit ($\sigma^{2}$) reaches to the values close to 1.5, showing the convergence of the refined profile with the observed patterns and confirming the quality of fit^53^. Cation distribution was obtained by taking into account the presence of both inverse and normal spinel structures. The nickel ferrite was modeled as complete inverse, while the zinc ferrite was considered as complete normal spinel. Furthermore, Raman spectroscopy was done using an XploRA PLUS (HORIBA, Japan) with a 785 nm laser line operated at room temperature. The microstructural characteristics were observed by a Mira 3 field-emission scanning electron microscope (TESCAN). An energy-dispersive detector with an accelerating voltage of 15 kV was used to depict the elemental composition. A vibrating sample magnetometer (Meghnatis Daghigh Kavir Co., Iran) was used to measure the magnetic properties at room temperature. The applied field was swept from − 10 to10 kOe. Electromagnetic properties, including permittivity and permeability spectra, were obtained using an 8722 ES network analyzer (Agilent/HP) in the wide frequency range of 1–18 GHz.

## Results and discussion

**Figure 2:**
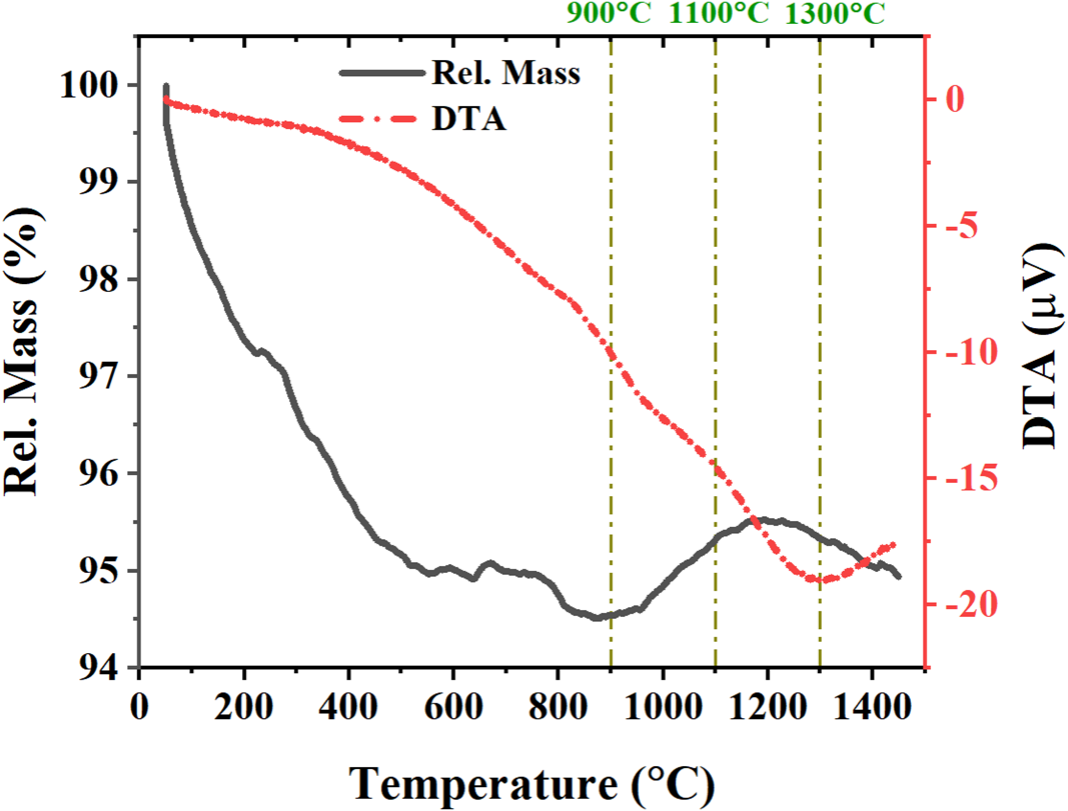
DTA/TG analysis results of the as-milled powders.

Figure 2 represents the DTA-TG plot of as-prepared powders. The initial mass reduction (about 5%) starts from ambient temperature up to 600 °C due to the elimination of physically absorbed water in most (3.5% up to 250 °C) and dehydroxylation and decomposition of residual organic compounds. Three critical temperatures in TG curve at 900, 1100, and 1300 °C were chosen for further thermal treatments of calcination and sintering where the main mass reduction occurs, spinel phase formation initiates, and sintering phase completes, respectively. Moreover, fluctuations of DTA curve at about 930, 1120, and 1280 °C were attributed to the mentioned critical temperatures, where the broad endothermic peak at 1200–1400 °C can also be assigned to the grain growth step of the sintering. Small fluctuations and negligible mass changes in the thermally analyzed sample illustrate the stability of the compound at higher temperatures^54^.

**Figure 3:**
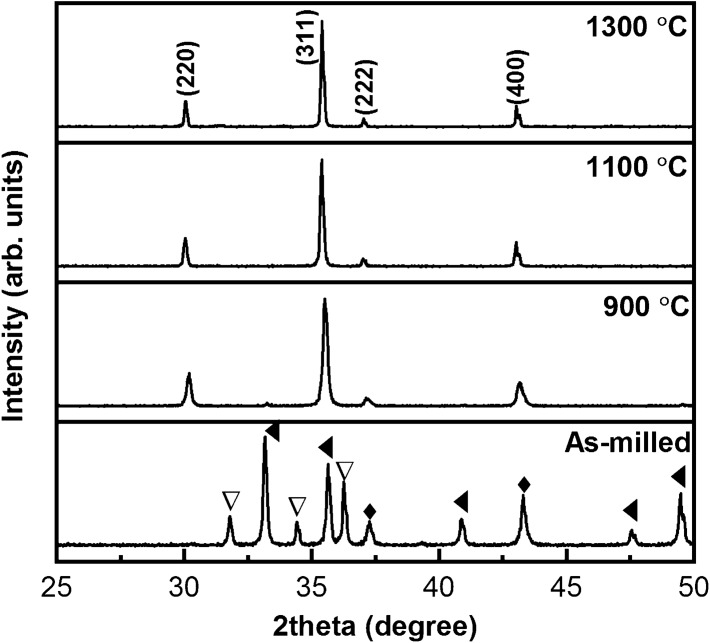
XRD patterns of the as-milled and calcined powders at 900 °C and the samples sintered at 1100 and 1300 °C (▽: ZnO, ◆: NiO, and ◄: Fe_2_O_3_).

Figure 3 shows the XRD patterns of the initial mixture, as-calcined Ni–Zn ferrite powder, and sintered samples. During the calcination process, the finely crushed NiO, ZnO, and Fe_2_O_3_ powders disappear by reacting the ZnO species with Fe_2_O_3_ to crystallize the zinc ferrite (ZnO.Fe_2_O_3_). After that, the NiO species slowly diffuses into zinc ferrite to produce NiZn ferrite (Ni_0.5_Zn_0.5_Fe_2_O_4_) powders^55,56^. Therefore, the chemical reaction with stoichiometric ratios can be written as $0.5{\text{NiO}} + 0.5{\text{ZnO}} + {\text{Fe}}_{2} {\text{O}}_{3} \to {\text{Ni}}_{0.5} {\text{Zn}}_{0.5} {\text{Fe}}_{2} {\text{O}}_{4}$. The diffraction peaks of the as-calcined powders and sintered samples at 2θ = 30.04°, 35.40°, 37.03°, and 43.03° are related to (022), (311), (222), (004) planes of the spinel structure of N_0.5_Zn_0.5_Fe_2_O_4_ phase (PDF2# 00-052-0278), respectively. Although a small peak of Fe_2_O_3_ phase was detected in the XRD pattern of calcined sample at ~ 33°, the absence of any other peaks at higher temperatures confirms the purity of sintered Ni–Zn ferrite samples. The lattice constant, density, cations distribution, dislocation density, and strain of the samples versus sintering temperature are shown in Table 1^57^. The lattice constant (a) decreases from 8.3892 to 8.3872 Å, while the density increases from 4.31 to 4.92 g cm^−3^ because of the reduction of crystal defects with the increase of sintering temperature. Distribution of cations show that the Zn cations are preferred to occupy tetrahedral (A) sites, while the Ni and Fe cations are distributed between tetrahedral (A) and octahedral [B] sites in which the fraction of Fe cations in (A) site as inversion coefficient decreases from 0.4 to 0.3 with the sintering temperature.

**Table 1:** The lattice parameter, theoretical density (ρ_XRD_), cation distribution, strain (ε), and dislocation density (δ).

Temperature (°C)	a (Å)	ρ_XRD_ (g cm^−3^)	Cation distribution	$\varepsilon \times 10^{ - 3}$	$\delta \times 10^{ - 3} \left( {{\text{nm}}^{ - 2} } \right)$
1100	8.3892	5.3486	(Ni_0.1_Zn_0.5_Fe_0.4_)_A_[Ni_0.4_Fe_1.6_]_B_O_4_	2.06	0.03
1300	8.3872	5.3525	(Ni_0.2_Zn_0.5_Fe_0.3_)_A_[Ni_0.3_Fe_1.7_]_B_O_4_	1.24	0.01

**Figure 4:**
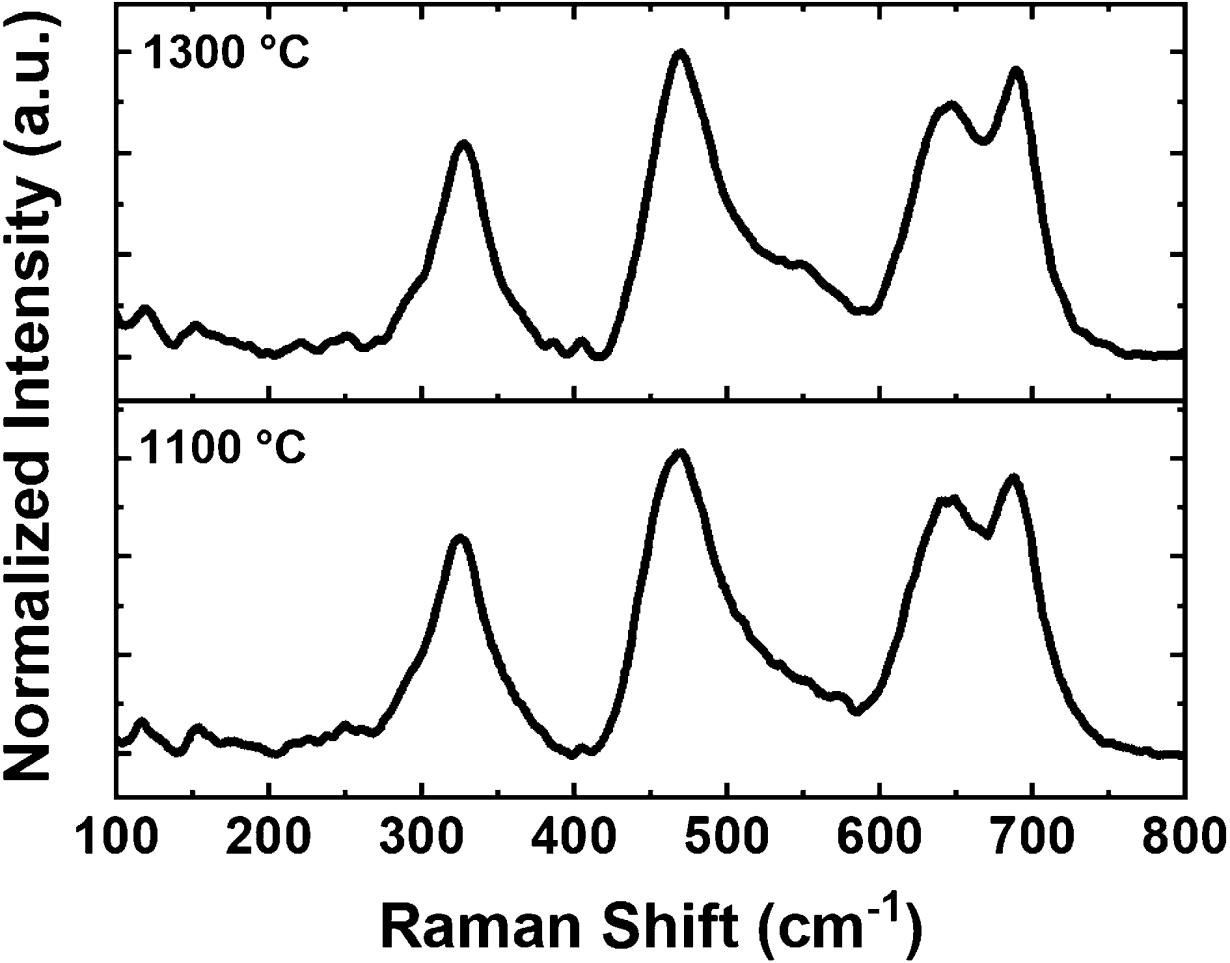
Raman spectra of the sintered samples.

Figure 4 shows the Raman spectra of the sintered samples at 1100 and 1300 °C. Small and broad peaks at about 210, 300, 460, 640, and 680 cm^−1^ are related to the five active Raman modes of T_2g_(1), E_g_, T_2g_(2), T_2g_ (3), and A_1g_^58^. Low Raman shifts revealed the presence of octahedral B-sties (BO_6_), while the observed peaks at about 640–700 cm^−1^ are attributed to oxygen motion in the tetrahedral A-sites (AO_4_) having A_1g_ mode Raman character^59,60^. This broad containing shoulder peak was also been assigned to the order–disorder effect of ions over A and B sites^61^. The related active Raman shifts presented in Table 2 confirms the formation of cubic spinel ferrite, Fd3m, in the sintered samples. Also, all Raman peaks blue shifted slightly toward higher wavenumber due to the increase in sintering temperature (to 1300 °C), where the crystal defects and strain reduced and consequent lower lattice parameter values were obtained^30^.

**Table 2:** Raman spectra peaks and related active modes.

Temperature	T_2g_ (1)	E_g_	T_2g_ (2)	T_2g_ (3)/shoulder peak	A_1g_
1100 (°C)	211	325	469	646	686
1300 (°C)	212	326	469	648	688

**Figure 5:**
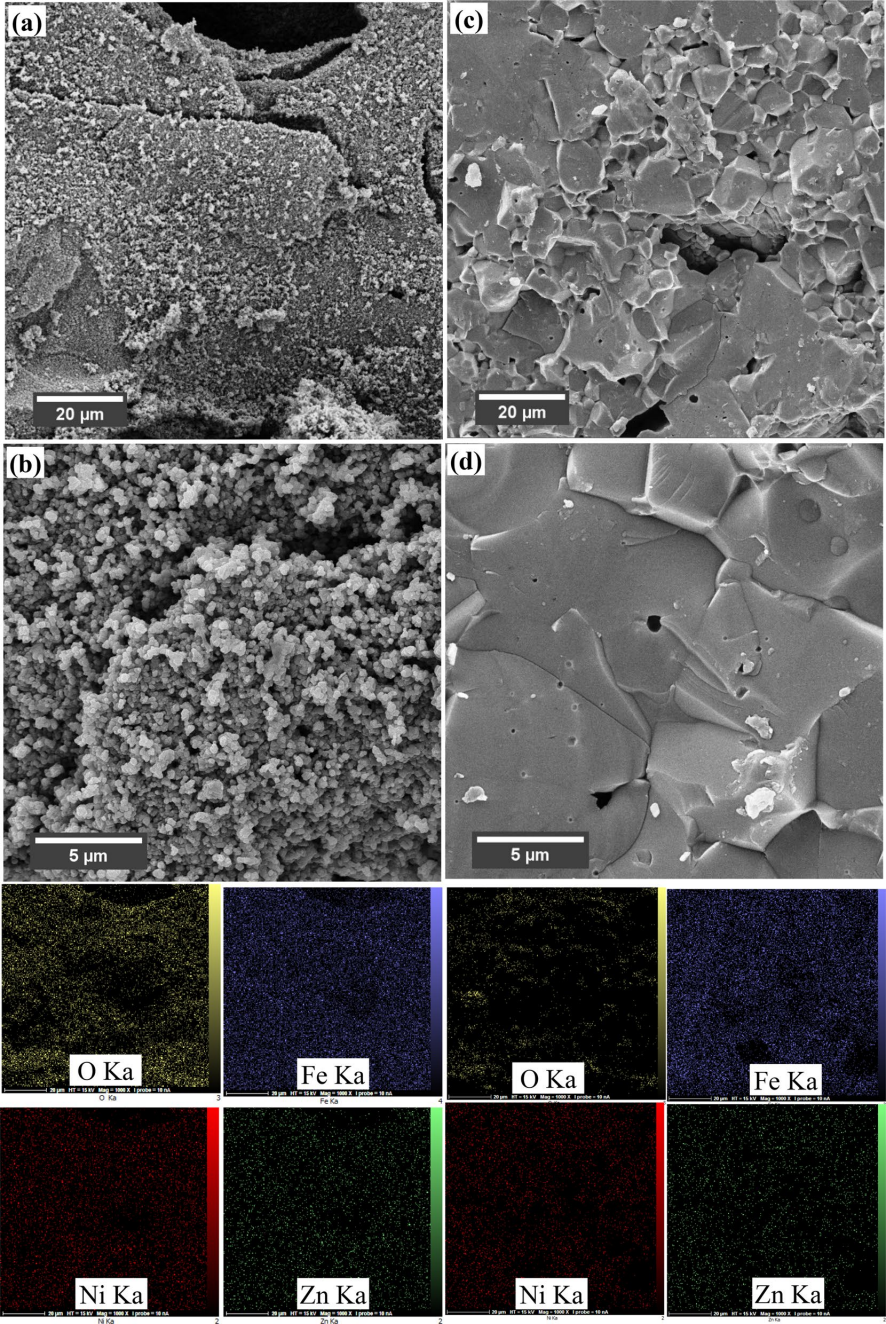
SEM micrographs and EDS maps of the samples sintered at (**a**, **b**) 1100 and (**b**, **c**) 1300 °C.

FESEM micrographs and EDS analysis of the sintered samples at 1100 and 1300 °C are presented in Fig. 5. The samples sintered at 1100 °C have small (~ 0.3 µm) and discrete grains, while the large grains (~ 10 µm) with smooth grain boundaries appear for sintering at 1300 °C. This may affect the magnetic and microwave absorption properties of the samples through the interface effects^62,63^. Also, it can be observed that the Ni–Zn ferrite particles cannot be grown and sintered at the lower temperature. Densification is chiefly related to grain boundary (GB) diffusion, while grain growth is ruled by GB migration. The abnormal grain growth at 1300 °C can be attributed to the small grain size of Ni–Zn ferrite powders, providing a large amount of activation energy for grain boundary migration. Some small pores are also trapped inside the ferrite grains because of the diffusion lag during the sintering process. Although some pores may be trapped, the resultant structure has lower defects and pores and therefore possess more strength comparing with non-sintered structure. Moreover, with the increase of sintering temperature from 1100 to 1300 °C, the density of samples increases from 4.31 to 4.92 g cm^−3^, respectively, which are 80.5 and % 91.9 of theoretical density (ρ_XRD_), confirming the decrease of pores and increase of the strength during sintering. Reddy et al.^64^ reported that the 0.5 atomic Zn ion enhanced the sintering and led to higher grain growth. Also, Mangalaraja et al.^65^ obtained the highest mechanical properties for the samples with Zn = Ni = 0.5 sintered at 1350 °C. The EDAX analysis shows the various elements are uniformly distributed in the grains and grain boundaries.

**Figure 6:**
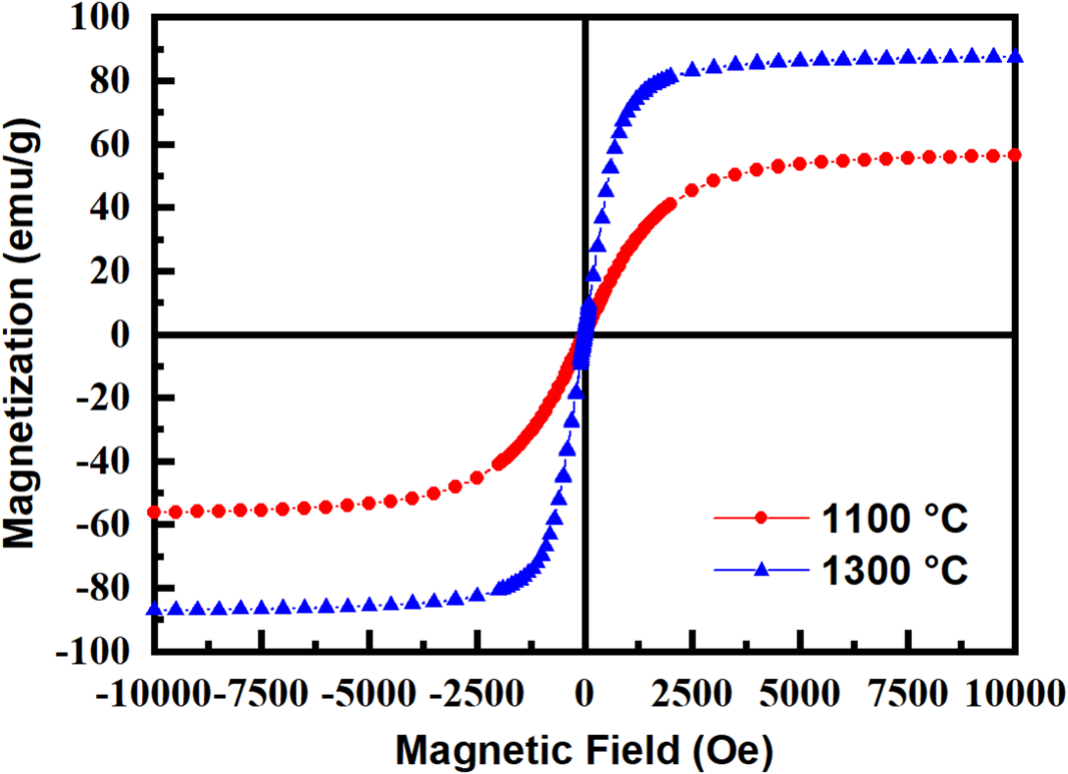
Magnetic hysteresis curves of the samples sintered at 1100 and 1300 °C.

Figure 6 compares the magnetization curve of the sintered samples. The samples sintered at 1100 °C have the saturation magnetization (Ms) of 56 emu/g with the coercivity (Hc) of 12 Oe, which the coercivity decreases to 4 Oe and the Ms increases to 87 emu/g for 1300 °C. The reduction of Hc can be attributed to the large grains, facilitating the magnetic domain wall motion. Also, the smooth interfaces here may decrease the Hc, according to the reports on the effect of interface roughness on increasing the Hc^63,66^. Aggarwal and Narang^67^ gained the highest Ms of about 71 emu/g and Hc of 35 Oe for the samples prepared by sol–gel followed by 8 h of heat treatment at 1000 °C. The same magnetization of 72 emu/g with a very low coercivity of 2 Oe was attained by Liu et al.^68^ at a higher sintering temperature of 1250 °C. The magnetization of spinel ferrites is mainly dependent on purity, crystallinity, and cations distribution between tetrahedral and octahedral sites^46,69^. In the collinear two sublattice model, the magnetic moments in the tetrahedral (M_A_) and octahedral (M_B_) sites mostly determined the final magnetization value of the samples rather than the A–A and B–B exchange interactions. The most |M_A_ − M_B_| absolute difference value leads to the highest Ms value of the ferrite samples. Zn as a non-magnetic ion could modify the amount of magnetization in the ferrite sample by inducing the magnetic cation migration from tetrahedral to octahedral sites. According to Neel’s theory, the high imbalance of magnetic ions such as Fe^3+^ and Ni^2+^ between (A) and [B] sites (Table 1) results in the high saturation magnetization for the sample sintered at 1300 °C. The higher imbalance of Fe^3+^ cations for higher sintering temperatures can be attributed to the higher cooling rate^70^ and crystal strains. Moreover, the high crystallinity and large grains are also beneficial for the increase of Ms at higher sintering temperatures.

**Figure 7:**
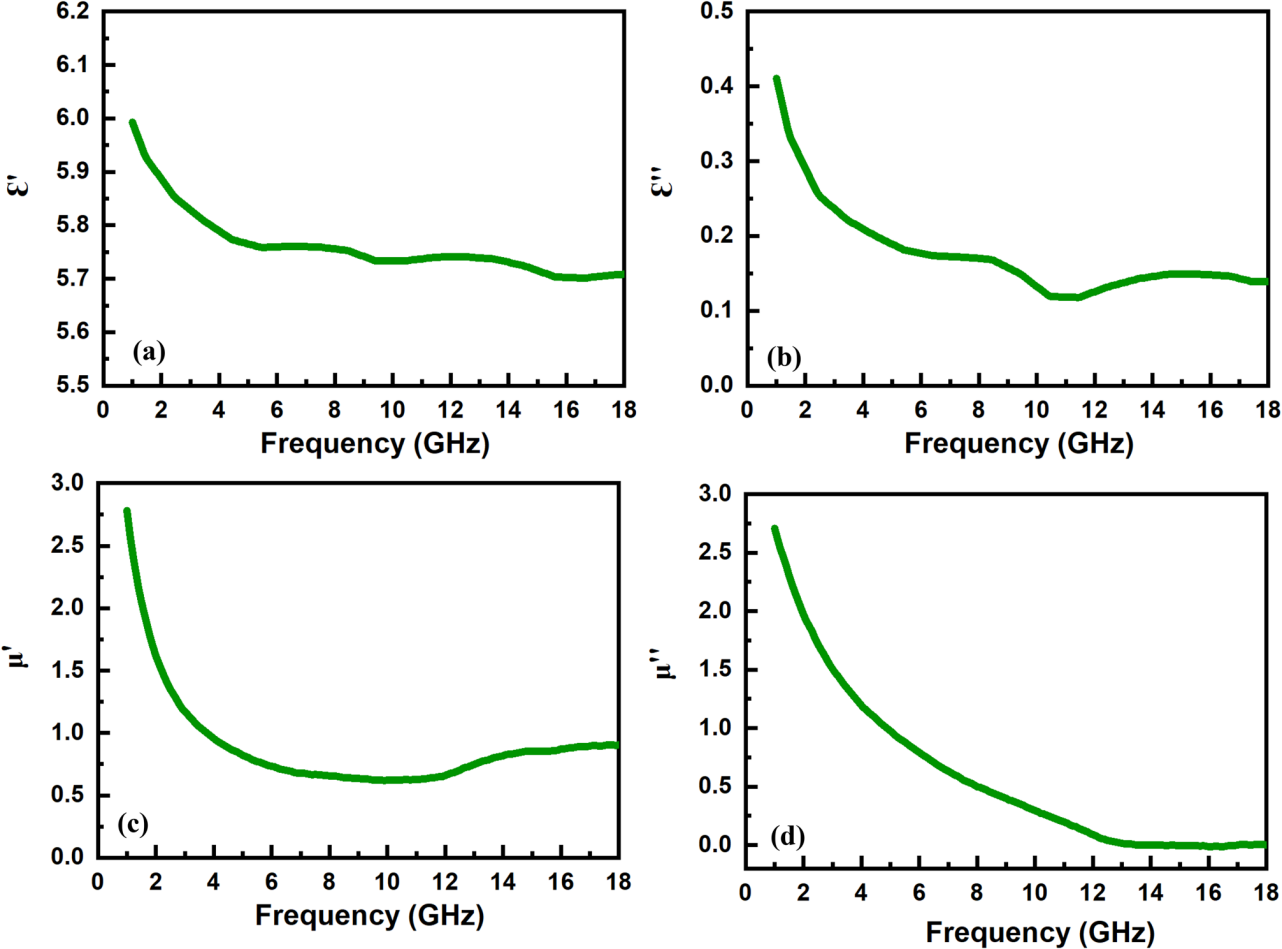
Permittivity and permeability spectra of the sample sintered at 1300 °C.

**Figure 8:**
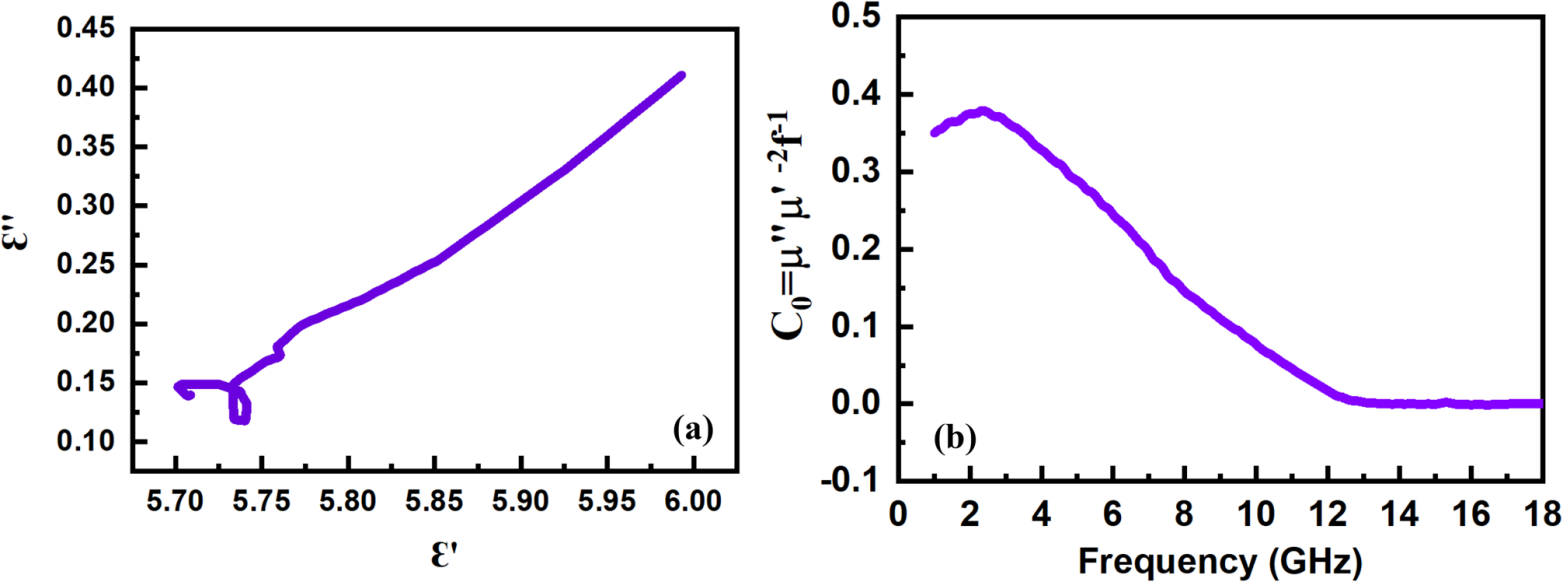
(**a**) Cole–Cole plot and (**b**) frequency dependence of μ″ (μ′)^−2^f^−1^ of the sample sintered at 1300 °C.

Figure 7 shows the permittivity (ε_r_ = ε′ − jε″) and permeability (µ_r_ = µ′ − jµ″) spectra of the sintered sample at 1300 °C. The real parts of spectra are related to the storage of electrical and magnetic energy, while the energy dissipation is shown by imaginary parts. The values of ε′ decrease versus frequency from 6 at 1 GHz to 5.7 at 18 GHz with an average value of about 5.8. The values of ε″ show two characteristic peaks at ~ 8.5 and 14.5 GHz over the 1–18 GHz range. The electrical energy of EM waves is dissipated by the conductivity and polarization mechanisms. The atomic, electronic, and dipolar polarizations are the main mechanisms of dielectric loss^71^. Among them, only dipolar polarizations occur in the microwave range due to the interfacial relaxations in which the accumulation of free charges at grain boundaries and defects of material enhances the dipoles^72–74^. The complex permittivity (ε_r_) can be effectively modeled by considering the Debye equation for dipolar polarization and $\frac{\sigma }{{\omega \varepsilon_{0} }}$ for conductivity losses as follows^75^:

1 $$\varepsilon_{r} = \varepsilon^{\prime} - j\varepsilon^{\prime\prime} = \mathop \sum \limits_{i} \left( {\varepsilon_{r\infty } + \frac{{\varepsilon_{r0} - \varepsilon_{r\infty } }}{{1 + \left( {j\omega \tau } \right)^{1 - \alpha } }}} \right)_{i} - j\frac{\sigma }{{\omega \varepsilon_{0} }}$$

where the first term describes the superposition of different relaxation mechanisms and the second one is related to the contribution of electrical conductivity $\left( {\frac{\sigma }{{\omega \varepsilon_{0} }}} \right)$ in which σ = σ_dc_ + σ_ac_. The ω = 2π*f* is the operating angular frequency, the values of ε_r0i_ and ε_r∞i_ are permittivity at low‐frequency and high‐frequency, respectively, τ_i_ is the relaxation time, and 0 < α_i_ < 1 is a non‐ideality empirical constant in *i*th relaxation mechanism. Large ferrite grains are partially conductive, whereas grain boundaries have poor conductivity. At high-frequency ranges, large grains prevail grain boundaries which were effective at low frequencies. Also, the Maxwell–Wagner double layer model expressed that ε′ is decreased and σ_ac_ is increased as frequency increased^76^. Therefore, the dispersive behavior of ε″ values with the frequency can be attributed to the higher contribution of AC conductivity (σ_ac_). Furthermore, the peaks of ε″ spectra are related to the contributed relaxations, such as interfacial polarization between the ferrite grains, as can be confirmed by the Cole–Cole plot (Fig. 8a)^77,78^.

Figure 7c and d show the real and imaginary parts of permeability versus frequency. The values of μ′ and μ″ decrease with the increase of frequency because of the magnetization relaxations^79,80^. The magnetic losses in the GHz region are mainly related to the eddy current and ferromagnetic resonance mechanisms^81,82^. The ferromagnetic resonances, including the natural resonance and wall resonance, occur in the MHz range for the soft magnetic NiZn spinel ferrite due to its low anisotropy field (H_A_)^83,84^. However, the natural resonance was observed at higher frequencies (GHz range) because of the exchange resonance^29,85,86^. On the other hand, the eddy current effect loss can be the main mechanism in the GHz region for spinel ferrites. The eddy current loss is dominant when the value of C_0_ = 2πt^2^σμ_0_ = μ″ (μ′)^−2^f^−1^ is independent of frequency^87^. As shown in Fig. 8b, the C_0_ values are constant at above 12 GHz, indicating the predominance of the eddy current loss.

The permittivity (ε_r_) and permeability (μ_r_) parameters can be used for the calculation of normalized impedance of absorber (Z_in_) relative to free space (Z_0_) and reflection loss *RL* (dB) versus frequency (*f*) and thickness (*t*), according to the transmission line theory as follows^88,89^:

2 $$Z_{in} {/}Z_{0} = \sqrt {\mu_{r} {/}\varepsilon_{r} } \tanh \left( {j\left( {2\pi ft{/}c} \right)\sqrt {\mu_{r} {\epsilon}_{r} } } \right)$$

3 $$RL\left( {{\text{dB}}} \right) = 20log\left| {\frac{{Z_{in} {/}Z_{0} - 1}}{{Z_{in} {/}Z_{0} + 1}}} \right|$$

**Figure 9:**
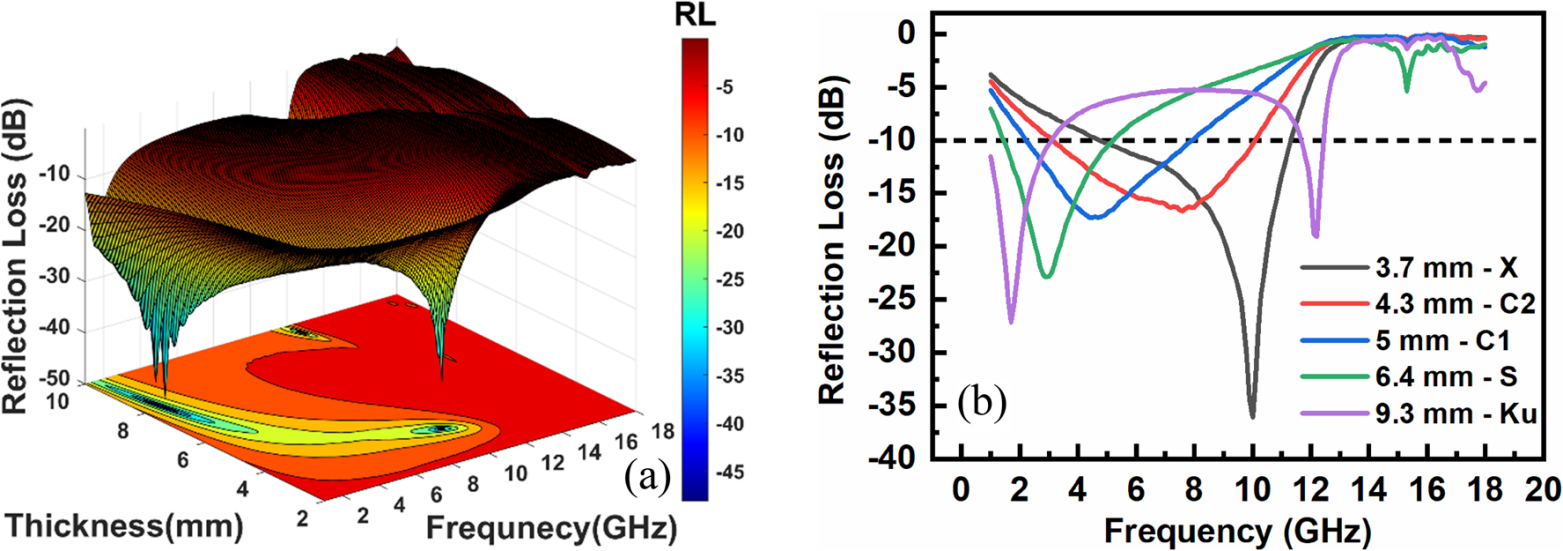
(**a**) Reflection loss (RL) plots showing the distribution of RL versus thickness and frequency and (**b**) RL plots for the optimum thickness in various bands.

**Figure 10:**
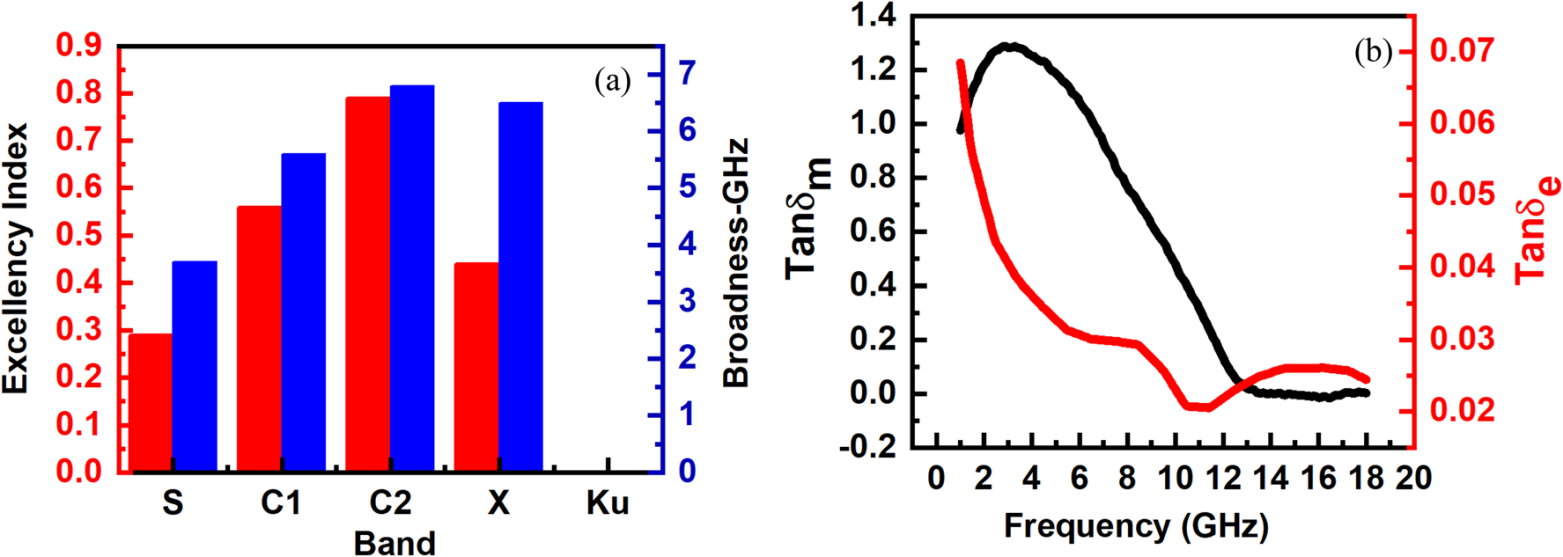
(**a**) PI index for various bands and (**b**) dielectric and magnetic loss versus frequency.

The dependency of reflection loss on the frequency and thickness is shown as a 3D contour plot in Fig. 9a. The higher magnetic and dielectric loss of EM waves by the absorber leads to a more negative RL because of the lower wave reflection from the absorber. High reflection loss up to − 48.1 dB is attained at the frequency range of 1–18 GHz, as shown in the 3D plot. Also, by data extraction from the plot, well-known Wireless LAN protocols of IEEE 802.11b, y, and j at ~ 2.4, 3.65, and 5 GHz can be fully suppressed by the NiZn ferrite absorber. Furthermore, the optimum matching thickness at each frequency band was determined for obtaining the maximum RL and wide effective bandwidth (bandwidth at − 10 dB), as shown in Fig. 9b. The sintered Ni–Zn ferrite showed the maximum RL of − 36.1 dB at the matching frequency of 10 GHz (X-band) in the matching thickness of 3.7 mm, having twice lesser reflection loss than that of Aggarwal and Narang^41^ reported at the same frequency band (− 17.54 dB at 9.62 GHz) and close to the Nd-doped nickel–zinc ferrite fabricated by Jiao et al.^90^ (− 37 dB at 8.4 GHz). For evaluating the current study microwave absorption performance with that of other researchers, Table 3 gives a comparison by representing the composition, fabrication method, minimum reflection loss (RL_min_), matching frequency (f_m_), effecting working bandwidth (EWB), and matching thickness (t_m_) of the similar Ni–Zn ferrite studies in the gigahertz region. The predominancy index (PI) as the ratio of effective bandwidth to matching thickness at various bands is shown in Fig. 10a^91^. The values of PI are 0.29, 0.56, 0.79, 0.44, and 0 for S, C1, C2, X, and Ku bands. The maximum effective bandwidth at the minimum matching thickness is for the C2 band. Mustaffa et al.^42^ reported the microwave absorption properties of MWCNT/Ni_0.5_Zn_0.5_Fe2O_4_ composite in the X-band and obtained the PI index of about 0.11 at the matching thickness of 3 mm. The energy of EM waves is consumed by both dielectric and magnetic losses, can be compared by loss factor (tanδ) versus frequency (Fig. 10b). It defines as the ratio of imaginary to real components of the complex permittivity and permeability spectra. The magnetic loss is dominant in the range of 1–12 GHz, while the dielectric loss at above 12 GHz. The magnetic loss can be attributed to the resonance mechanisms at the frequency of 1–12 GHz and the eddy current loss at 12–18 GHz.

**Table 3:** Microwave absorption performance of NZF tile EM absorber (current study) compared to other GHz-region NZF absorbers published articles.

Composition	Fabrication method	RL_min_ (dB)	f_m_ (GHz)	EWB (GHz)	t_m_ (mm)	References
NZF	Solid-State followed by sintering @ 1300 °C	− 36.1	10	6.7	3.7	Current Work
NZF	Solid-State followed by sintering @ 1300 °C	− 16.9	7.6	7.1	4.3	Current Work
NZF	Solid-State followed by sintering @ 1300 °C	− 48.1	2.1	2.7	8	Current Work
Nd-doped NZF/PANI	Sol–gel propagate combustion (SPC)	− 37.4	8.4	4.9	4	^90^
NZF/E-glass/epoxy	Sol–gel autocombustion	− 33 dB	9.6	2.4	4	^92^
NZF/epoxy	Sol–gel followed by annealing @ 1100 °C	− 33.64	10.2	8	4.1	^51^
Co-Zr-doped NZF	Sol–gel citrate followed by sintering @ 1000 °C	− 44.92	9.2	2.94	4.25	^93^
NZF	followed by sintering @ 1000 °C	− 17.54	9.6	3.1	3.5	^41^
PPy/NZF/paraffin	Oxidative polymerization followed by calcination @ 700 °C	− 30 dB	10.9	3.6	3 mm	^94^
MWCNT/NZF	Mechanical alloying followed by sintering @ 1200 °C	− 19.34	8.46	1.24	3 mm	^42^
NZF/SFO/paraffin	Sol–gel self-propagating followed by calcination @ 1000 °C	− 47 dB	6.2	6.4	3.5 mm	^95^
Co-doped NZF/GN/paraffin	Hydrothermal	− 53.5	9.6	4.8	3.0 mm	^78^
NZF nanofiber/paraffin	Electrospinning	− 14 dB	10.9	4.4	4 mm	^35^
NZF/TPNR	Precipitation	− 38.3	9.6	7	7 mm	^96^

The total contribution of dielectric and magnetic attenuation ability can be evaluated by the attenuation constant (α), which is defined by the following equation^97^:

4 $$\alpha = \frac{\sqrt 2 \pi f}{c}\sqrt {\left( {\mu^{\prime\prime}\varepsilon^{\prime\prime} - \mu^{\prime}\varepsilon^{\prime}} \right) + \sqrt {\left( {\mu^{\prime\prime}\varepsilon^{\prime\prime} - \mu^{\prime}\varepsilon^{\prime}} \right)^{2} + \left( {\mu^{\prime}\varepsilon^{\prime\prime} + \mu^{\prime\prime}\varepsilon^{\prime}} \right)^{2} } }$$

**Figure 11:**
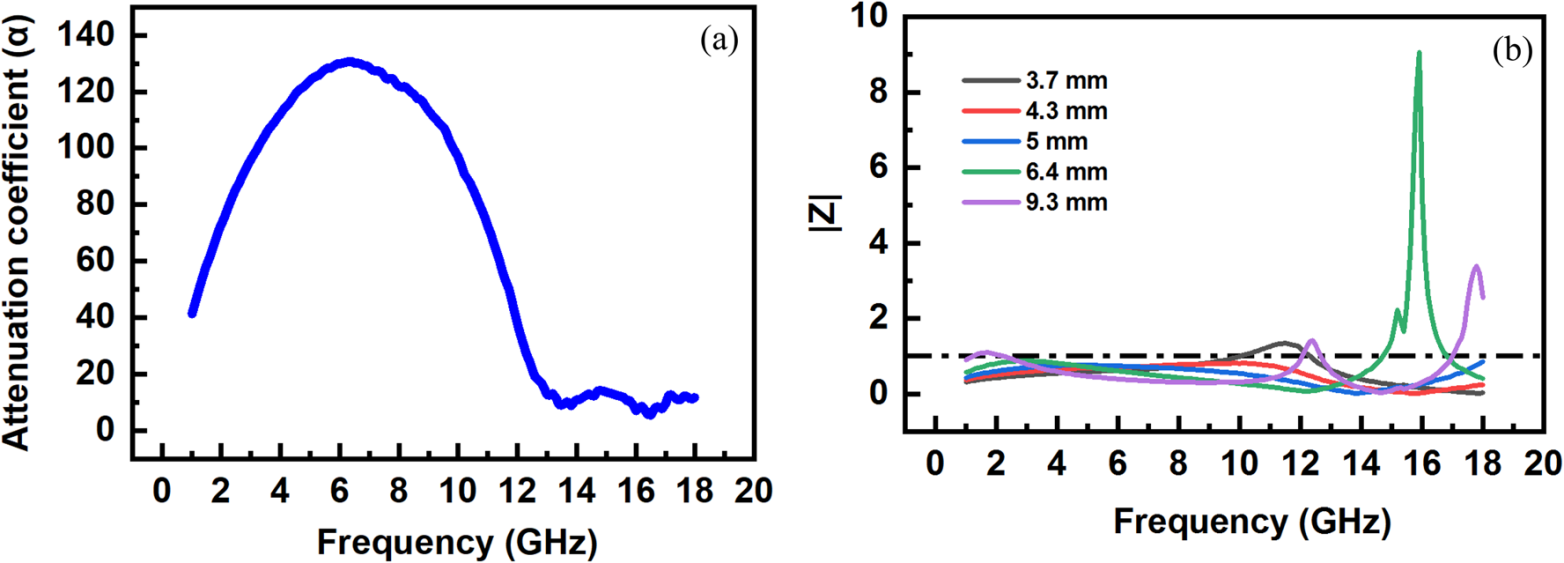
(**a**) Attenuation values versus frequency, and (**b**) normalized impedance versus frequency and thicknesses.

Figure 11a shows a broad peak in the frequency range of 2–12 GHz in which the increase of α values is related to the high contribution of dielectric and magnetic losses, while the strong reduction of dielectric loss results in the decrease of α values at higher frequencies. The incident EM waves can penetrate the absorber with the minimum reflection when the input impedance is well-matched with free space (Z_0_). In other words, the modulus of normalized impedance, $\left| Z \right| = \left| {\frac{{Z_{in} }}{{Z_{0} }}} \right|$, should be approached to 1^98,99^. Figure 11b presents the normalized impedance at different matching thicknesses. It can be found that the values of |Z| are enough close to 1 at higher thicknesses. Therefore, the EM waves can be easily penetrated to the absorber and attenuated by magnetic and dielectric loss mechanisms.

## Conclusion

Due to the emergence of wireless communications, the utilization of electromagnetic absorber materials is mandatory to reduce electromagnetic interference and bring electromagnetic compatibility for electronic devices. In this research, Ni–Zn ferrite (Ni_0.5_Zn_0.5_Fe_2_O_4_) bulk samples were fabricated to use as a tile microwave absorber material in the wide 1–18 GHz frequency range. The sample sintered at 1300 °C had a large grain size (~ 10 µm) with smooth grain boundaries. In addition, higher sintering temperature led to appropriate magnetic properties, including high saturation magnetization of 87 emu/g and low coercivity of 4 Oe. 3D reflection loss plot showed the distribution of loss over the 1–18 GHz frequency range in which high reflection losses up to − 48.1 dB were obtained at certain low and high frequencies. The optimized condition by the preference of working bandwidth was in the range of ~ 3 to 10.1 (7.1) GHz for the sample with the matching thickness of 4.3 mm with RL of − 16.9 dB, while by RL preference, the minimum value of − 36.1 dB was achieved at the thickness of 3.7 mm with the effective absorption bandwidth of 6.7 (4.7–11.4) GHz. The absorption performance of the sintered Ni–Zn ferrite was related to easy penetration of EM waves and further attenuation by magnetic and dielectric loss mechanisms. The magnetic loss was attributed to the resonance mechanism, while the interfacial polarization was responsible for the dielectric loss.
